# Supplementary material for: Epidural Stimulation and Resistance Training (REST-SCI) for Overground Locomotion After Spinal Cord Injury: Randomized Clinical Trial Protocol
Source: J Clin Med. 2025 Mar 8;14(6):1829. doi: 10.3390/jcm14061829 (PMC11942695; doi:10.3390/jcm14061829)
Supplement: Supplementary file 1 [file jcm-14-01829-s001.zip › Supplementary Videos R5.pdf]

SUPPLEMENTARY VIDEO LEGENDS

Supplementary Video S1. On the same day as the temporary percutaneous implantation (Day 1), participant 0773 demonstrated minimal ability to flex his right hip or move in a side lying position with spinal cord epidural stimulation (SCES) turned off. EMGs for abdominal and right leg muscles for this video are shown in the red-shaded portion of supplementary figure 6.

Supplementary Video S2. In contrast to when SCES was off, participant 0773 could flex his right hip and produce active movement with SCES turned on. EMGs for abdominal and right leg muscles for this video are shown in the green-shaded portion of supplementary figure 6.

Supplementary Video S3. On the day after temporary implantation (Day 2), participant 0773 could voluntary flex his left leg in a supine position. Attempts to maintain the contraction and hold his leg in the flexed position are also demonstrated.

Supplementary Video S4. Participant 0773 stepping in parallel bars using SCES. Details of the SCES configuration are in Figure 1. The participant stepped with no external assistance from study staff, and could not step with SCES off.

Supplementary Video S5. Participant 0773 stepping overground with a four-point walker using SCES. Details of the SCES configuration are in Figure 1. The participant stepped with no external assistance from study staff, and could not step with SCES off.
